# Preliminary Findings of a National Survey of Black ADRD Family Caregivers

**DOI:** 10.1093/geroni/igab046.1079

**Published:** 2021-12-17

**Authors:** Sheria Robinson-Lane, Florence Johnson, Nicholas Mazzara, Kayla DeMarco, Ivaylo Dinov

**Affiliations:** 1 University of Michigan School of Nursing, Ann Arbor, Michigan, United States; 2 University Of Michigan School of Nursing, Ann Arbor, Michigan, United States

## Abstract

The National Caregiver Survey aims to capture a representative sample of Black Alzheimer’s disease and/or related dementias (ADRD) family caregivers who are 55+ to better understand the relationships between adaptation to caregiving, coping, and health. Following targeted social media marketing, ADRD family caregivers (n=60) completed an electronic survey capturing over 200 data elements. Analysis was completed using Spearman correlation coefficients. Preliminary results suggest that 55% of participants were hypertensive (n=33) and 27% (n=16) had diabetes. Participants were generally overweight with an average BMI of 29. 28% (n=17) of the sample were smokers. A negative correlation was identified between the level of care needs of the recipients (IADLs) and alcohol use (p=0.037). There was also a correlation between identifying positive aspects of caregiving and adaptive coping (p=0.045). Caregiver support programs should facilitate development of effective coping strategies for new family caregivers, with particular attention on smoking cessation, brain-healthy diet, and exercise.

